# Effect of the medical emergency team on long-term mortality following major surgery

**DOI:** 10.1186/cc5673

**Published:** 2007-01-29

**Authors:** Daryl Jones, Moritoki Egi, Rinaldo Bellomo, Donna Goldsmith

**Affiliations:** 1 Australian and New Zealand Intensive Care Research Centre (ANZIC-RC), Department of Epidemiology and Preventive Medicine, Monash University, Commercial road Melbourne, Victoria, 3004, Australia; 2 Department of Anesthesiology and Resuscitology, Okayama University Medical School, 2-5-1 Shikata city, Okayama, 700-8525, Japan; 3 Department of Intensive Care and Department of Medicine (Melbourne University) Austin Hospital, Studley Road, Heidelberg, Melbourne, Victoria, 3084, Australia

## Abstract

**Introduction:**

Introducing an intensive care unit (ICU)-based medical emergency team (MET) into our hospital was associated with decreased postoperative in-hospital mortality after major surgery. The purpose of the present study was to assess the effect of the MET and other variables on long-term mortality in this patient population.

**Methods:**

We conducted a prospective, controlled, before-and-after trial in a University-affiliated hospital. Participants included consecutive patients admitted for major surgery (surgery requiring hospital stay > 48 hours) during a four month control phase and a four month MET phase. The intervention involved the introduction of a hospital-wide ICU-based MET service to evaluate and treat ward patients with acutely deranged vital signs. Information on long-term mortality was obtained from the Australian death registry. The main outcome measure was patient mortality at 1500 days. Data on patient demographics, surgery undertaken and whether the surgery was scheduled or unscheduled was obtained from the hospital electronic database. Multivariable analysis was conducted to determine independent predictors of 1500-day mortality.

**Results:**

There were 1,369 major operations in 1,116 patients during the control period and 1,313 operations in 1,067 patients during the MET (intervention) period. Overall survival at 1500 days was 65.8% in the control period and 71.6% during the MET period (*P *= 0.001). Patients in the control phase were statistically less likely to be admitted under orthopaedic surgery, urology and faciomaxillary surgery units, but more likely to be admitted under cardiac surgery or neurosurgery units. Patients in the MET period were less likely to undergo unscheduled surgery. Multivariable analysis revealed that age, unscheduled surgery and admission under thoracic surgery, neurosurgery, oncology and general medicine were independent predictors of increased 1500-day mortality. Admission during the MET period was also an independent predictor of decreased 1500-day mortality (odds ratio 0.74; *P *= 0.005).

**Conclusion:**

Introduction of a MET service in a teaching hospital was associated with increased long-term survival even after adjusting for other factors that contribute to long-term surgical mortality.

## Introduction

Serious adverse events (SAEs) are common among patients admitted to hospital [1]. A review of 30,121 medical records in New York State showed that SAEs affected nearly 4% of all admissions, of which 13.6% led to death [2]. Similar findings have been reported in Australia [3], Canada [4] and the UK [5], demonstrating that this is a worldwide problem. In a study of patients undergoing major surgery in our hospital, 16.9% suffered SAEs and 7.1% died [6].

Cardiac arrests and SAEs in hospital patients are typically not sudden or unexpected. Several studies have demonstrated that these events are heralded by derangements of commonly measured vital signs during the preceding 24 hours [7-9]. Medical emergency teams (METs), an example of a Rapid Response System (RRS), have been introduced into hospitals to identify, review and treat at-risk patients during the early phase of deterioration. The hypothesis underlying this approach is that early intervention in the course of deterioration improves outcome.

In a previous study [10] we demonstrated that introducing a MET service into our hospital was associated with decreased postoperative SAEs, postoperative mortality and mean duration of hospital stay. However, this study only reported on postoperative mortality to the point of hospital discharge. Furthermore, it did not account for possible confounders that might have contributed to the observed outcome differences. The aim of the present study was to assess the effect of introducing a MET service on long-term survival (to 1500 days, or 4.1 years) in a cohort of patients undergoing major surgery at our hospital. In addition, we assessed patient, procedure and system related variables that might also have influenced long-term postoperative survival.

## Methods

### Ethics considerations

We obtained Hospital Human Research Ethics Committee approval for implementation of the MET and for collection of data related to the study. The need for informed consent was waived by the Hospital Human Research Ethics Committee.

A separate ethics approval was obtained from the Australian Registry of Deaths for permission to follow up and cross-reference outcomes in our cohort of patients with the Australian Registry of Deaths, which records the deaths of all Australian citizens.

### The Hospital

Austin Health is a teaching hospital of the University of Melbourne. It has two campuses located in the north-east of Melbourne, a city with a population of nearly 4 million. One campus (400 beds) receives all acute admissions and the other caters for aged care and rehabilitation admissions. The acute care campus admits approximately 60,000 patients per year and is the campus where this study was conducted. The acute care campus has 21 ICU beds that admit approximately 1,800 patients per year. The ICU operates according to the 'closed' ICU model, where only ICU physicians can prescribe treatment.

### Preintervention rapid response team structure

Before the introduction of the MET, the hospital rapid response team was based on the traditional cardiac arrest team concept. Cardiac arrest team members carried pagers that were activated during the 'code blue' call. All wards are equipped with resuscitation trolleys containing resuscitation drugs and semi-automated defibrillators. The cardiac arrest team included a cardiology fellow, an intensive care fellow, a coronary care nurse and the receiving medical unit fellow of the day. This cardiac arrest response system remained unaltered throughout the study.

### The medical emergency team

Any member of hospital clinical staff (including nurses, physiotherapists, social workers, speech therapists, residents and members of senior medical staff) could activate the MET. The members of the MET included the duty intensive care fellow, a designated intensive care nurse and the receiving medical fellow. An ICU specialist was available to attend, if requested, from 08:00 until 20:00 hours. After hours, an intensive care specialist was available within 15 to 30 minutes for attendance if required. The criteria for MET activation were available in the form of a large red poster that was displayed prominently in each ward. Specifically, if any of the following conditions were present, staff were instructed to call 7777 and ask for the MET: staff member is worried about the patient; acute change in heart rate to < 40 or > 130 beats/minute; acute change in systolic blood pressure to < 90 mmHg; acute change in respiratory rate to < 8 or > 30 breaths/minute; acute change in pulse oximetry saturation to < 90% despite oxygen administration; acute change in conscious state; and acute change in urine output to < 50 ml in four hours.

The MET was activated by a pager call and by a public announcement internal communication call saying 'medical emergency team to ward X'. The MET was equipped with an emergency pack containing drugs and equipment needed for resuscitation and endotracheal intubation. After a MET call, if the patient was not admitted to ICU, then the MET visit was considered a formal consult, the parent unit was contacted, and concerns, advice and suggestions were verbally communicated and recorded in the patient's chart.

### Study design

The study design was that of a prospective controlled before-and-after intervention trial. All patients admitted to hospital who had major surgery were considered participants. Major surgery was defined as any operation associated with a hospital stay longer than 48 hours. In the present study we assessed the long-term mortality of the cohorts of patients reported in the original publication [10]. The follow-up time of 1500 days (4.1 years) represented the longest follow-up period for the MET (intervention) study cohort at the time of data acquisition from the Australian Registry of Deaths. To maintain consistency between the MET and control periods, outcome data were censored at this time.

### Study periods

The 'before' period was a four month period (control period) encompassing 1 May 1999 to 31 August 1999 (winter), during which outcome measures were studied under normal operating conditions of the hospital. This period was followed by a preparation and education period (1 September 1999 to 31 August 2000) to allow the introduction of the MET [10-12]. During this period the concept of the MET was presented in the form of lectures and tutorials to hospital administration, nursing staff and paramedical personnel (physiotherapists and speech therapists). Extensive and repeated presentations and discussions were held with all members of medical staff. Objections were raised and addressed at these meetings. The MET was then implemented and a run-in period of two months was allowed. This was done to ensure that there were no logistic or political problems with its implementation and that all members of hospital staff would become familiar with its use.

The 'after' period was the following four month period (intervention period) encompassing 1 November 2000 to 28 February 2001 (spring and summer) during which the outcome measures were studied under the new (availability of MET) operating conditions of the hospital.

### Patients

The analysis included all patients who had undergone in-patient surgery during the study period and who remained in hospital for 48 hours or more after surgery. The 48-hour limit was used to exclude patients having day surgery or minor procedures who were not expected to be at risk for SAEs.

### Data collection

We collected baseline demographical data (patient age and sex, procedure undergone) as well as hospital systems data (surgical specialty of admission, scheduled or unscheduled status of surgery). Information on long-term outcome was obtained from the Australian Registry of Deaths.

The number of procedures in each of 83 operative categories for both the control period and the intervention phase was collated to allow comparison. For the purposes of multivariable analysis, these 83 operative categories were then grouped into 37 operation clusters (labeled 1 to 37; Table 1). All collation and grouping was performed by a single investigator (DJ) who was blinded to patient outcome. Similarly, the investigator performing outcome analysis (ME) was blinded to the classification of the operation clusters (which were labeled 1 to 37) and the admitting unit.

**Table 1:** Characteristics of operation clusters 1 to 37

**Operation cluster**	**Operations contained within the cluster**
1. Open heart/thoracic aortic surgery	Aortic valve surgery with/without CABG surgery
	Aortic and mitral valve surgery
	Mitral valve surgery with/without CABG surgery
	Thoracic aortic repair/replacement
	CABG surgery and ventricular surgery
	CABG surgery and CEA

2. CABG surgery	CABG surgery

3. Resective thoracic surgery	Reoperation after cardiac surgery
	Sternal wound repair/closure
	Pericardial surgery
	Lobectomy or wedge resection
	Insertion of Denver shunt
	Lung biopsy
	Mediastinal surgery
	Thoracic surgery other

4. Pleural surgery	Pleurodesis
	Pleural decortication
	Drainage of empyema

5. Bronchoscopy	Brochoscopy (including laser and stent)

6. Upper gastrointestinal surgery	Oesophageal surgery
	Gastric resection/binding

7. Intestinal endoscopy/insertion of	Oesophagoscopy (including laser/stent)
feeding tube	Endoscopy
	Insertion of feeding tube

8. Appendicectomy	Appendicectomy

9. Hepatobilary or pancreatic surgery	Gall gladder/biliary surgery
	ERCP/bile duct manipulation
	Pancreatic/spleen resection
	Liver resection/portocaval shunt

10 Breast surgery	Breast surgery

11. Bowel surgery/hernia repair	Bowel resection/stoma formation or reversal
	Hernia repair

12. Head and neck, facial surgery	Thyroid/parathyroid surgery
	Head and neck resection
	Nasal surgery
	Facial surgery
	Tracheostomy surgery

13. Wound debridement/mass biopsy	Wound debridement/mass biopsy

14. Haemorroid/perianal surgery	Haemorroid/perianal surgery

15. Laparotomy other	Tenkoff catheter insertion
	Laparotomy other

16. Hip fracture	Hip replacement/dynamic hip screw

17. Joint aspiration/lavage	Joint aspiration/lavage/scope

18. Spinal fusion/laminectomy	Spinal fusion/laminectomy

19. Amputation	Amputation

20. Limb fracture	Repair fracture upper limb
	Repair fracture lower limb

21. Orthopedic other	Joint reconstruction/relocation
	Removal of prosthesis
	Tendon repair

22. Vascular bypass or	Lower limb vascular bypass
endarterectomy/fistula surgery	Upper limb vascular procedure
	Carotid endarterectomy
	Formation/exploration of fistula

23. Abdominal aortic aneurysm	Abdominal aortic aneurysm repair

24. Other vascular surgery	Renal transplant
	Varicose veins surgery

25. Cerebral aneurysm clipping	Aneurysm clipping

26. Draining ICH/abscess	Removal of ICH or AVM
	Drainage of intra-cranial abscess

27. Cerebral lobectomy/tumour resection	Removal of brain tumour
	Temporal lobectomy

28. Neurosurgery other	Insertion of spinal catheter
	Insertion/removal of VP shunt
	Neurosurgical other

29. Plastic surgical procedure	Hand surgery
	Removal of skin cancer and split skin graft
	Wound debridement and split skin graft
	Flap formation/reconstruction

30. Liver transplant	Liver transplant

31. Anaesthetic procedure	Elective direct current reversion
	Insertion of invasive lines
	MRI
	Other

32. Cystoscopy	Cystoscopy with/without transurethral resection of
	bladder tumour

33. Transurethral resection of prostate	Transurethral resection of prostate

34. Open prostatectomy	Open prostatectomy

35. Nephrectomy	Nephrectomy

36. Urology other	Cystectomy and ileal conduit formation
	Urolithiasis surgery

37. Gynaecological procedure	TAH and/or oophorectomy
	Dilatation and curretage
	Gynaecology other

AVM, arteriovenous malformation; CABG, coronary artery bypass graft; CEA, carotid end-arterectomy; ERCP, endoscopic retrograde cholangiopancreatography; ICH, intracerebral haemorrhage; MRI, magnetic resonance imaging; TAH, total abdominal hysterectomy; VP, ventriculoperitoneal.

### Outcome measures

The primary outcome measure for the study was the time to death (in days) from the date of admission. When performing multivariate logistic regression analysis, vital status at 1500 days was used as the dependent variable.

### Statistical analysis

Computerized statistical packages were used for data analysis and descriptive statistics (Statview [Abacus Inc., Berkeley, CA, USA] and SPSS 12.0 [SPSS Inc, Chicago, IL, USA]). Descriptive data are presented as mean ± standard deviation. Comparisons of nominal data for differences in proportions between the two study periods were performed using χ^2 ^or Fisher's exact test.

Cumulative mortality was determined using the Kaplan-Meier product limit method of survival estimation, and comparison of survival of patients in the MET and control periods was performed using the log-rank test, censoring survival at 1500 days.

We also performed multivariate logistic regression analysis using age (in ten year intervals), sex, unscheduled surgery, unit of admission, operation cluster (1 to 37) and MET period as independent variables, and vital status at 1500 days as the dependent variable. A forward stepwise elimination process was then used to remove covariates whose multivariate *P *value was > 0.10. The final model contained all predictors of mortality with a multivariate *P *< 0.10. In all multivariate logistic regression analyses, we sought to assess the following: the discrimination of the model with the percentages of appropriately classed patients in the final model; the calibration of the model with Hosmer-Lemeshow test; and the role of multicollinearity with the variance inflation factor. Every variance inflation factor was less than 5, indicating absence of severe multicollinearity.

For all statistical analysis, *P *< 0.05 was considered statistically significant.

## Results

### Baseline characteristics of the patient cohorts

During the control period, 1,369 procedures were conducted in 1,116 patients, and during the MET period 1,313 procedures were conducted in 1,067 patients (Table 2). The average age and proportion of female patients in the two periods was similar. Patients in the control period were statistically more likely to be admitted under units for cardiac or neurosurgery, and less likely to be admitted under units for orthopaedic surgery, urology, and ear nose and throat/faciomaxillary surgery (Table 2).

**Table 2:** Comparison of the demographics and allocation units for patients admitted during the control and MET periods

	Control period	MET period	*P *value
Number of patients	1116	1067	-
Age average (standard deviation)	60.8 (19.7)	60.1 (19.5)	0.46
Percentage female	41.52	42.61	0.49
Number of procedures	1369	1313	
Procedures per parent unit			
Cardiac surgery^a^	188	141	0.04
Thoracic surgery	141	117	0.22
General surgery/colorectal	288	313	0.08
Orthopaedic surgery^a^	253	289	0.02
Vascular surgery	160	132	0.17
Haematology	5	1	0.22
Neurosurgery^a^	147	112	0.05
Plastic surgery	77	84	0.40
Spinal injury unit	3	5	0.50
Liver transplant unit	28	15	0.06
Nephrology	8	2	0.11
Cardiology	4	4	>0.99
Oncology	6	2	0.29
Urology^a^	23	48	0.001
Gynaecology	7	12	0.21
Paediatric surgery	11	8	0.55
General medicine	10	3	0.09
ENT/faciomaxillary surgery^a^	9	24	0.005

^a^Indicates statistically significant differences in the proportion of admissions. ENT, ear, nose and throat; MET, medical emergency team.

### Differences in surgical procedures performed in the control and MET periods

Patients admitted during the MET period were less likely to undergo unscheduled surgery than those admitted during the control period (Table 3). In addition, patients admitted in the MET period were less likely to undergo valvular cardiac and aortic arch surgery, hepatobiliary, pancreatic, or splenic resection, vascular bypass and fistula surgery, and certain forms of neurosurgery (Table 3). In contrast, patients admitted during the MET period were more likely to undergo certain forms of orthopaedic and urological surgery (Table 3).

**Table 3:** Differences in the nature of operation clusters for patients admitted in the control and MET periods

	Control period	MET period	*P *value (OR, 95% CI)
Nonscheduled surgery	674	563	<0.0001 (0.66, 0.57–0.77)
Cardiac/thoracic aortic surgery (not CABG surgery)	73	41	0.001 (0.52, 0.35–0.77)
Hepatobiliary, pancreatic, and splenic surgery	96	74	0.04 (0.72, 0.52–0.99)
Orthopaedic 'other'	26	57	0.001 (2.15, 1.34–3.44)
Vascular bypass/fistula surgery	95	58	0.0008 (0.57, 0.41–0.79)
Intracranial haemorrhage/abscess drainage	29	9	0.0007 (0.29, 0.14–0.62)
Neurosurgery 'other'	42	27	0.04 (0.61, 0.37–0.99)
Anaesthesia related	12	26	0.03 (2.1, 1.05–4.20)
Urology 'other'	4	23	0.0004 (5.60, 1.92–16.2)

The characteristics of the operation clusters are outlined in Table 1. CABG, coronary artery bypass grafting; CI, confidence interval; MET, medical emergency team; OR, odds ratio.

### Differences in long-term mortality of patients admitted during the control and MET periods

**Figure 1:**
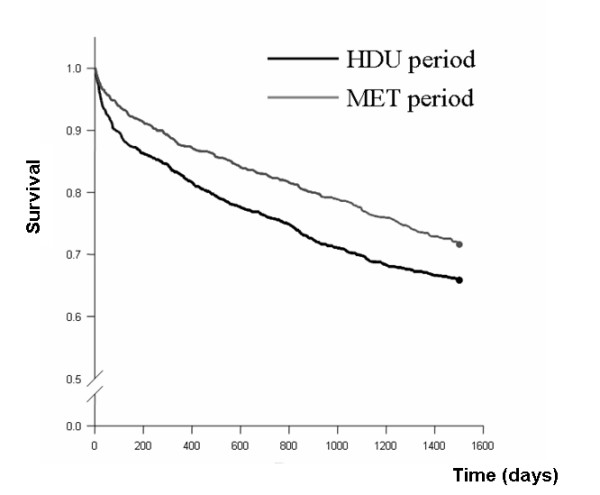
Kaplan-Meier survival curves for patients admitted during control and MET periods to 1500 days. MET, medical emergency team.

Patients admitted during the MET period had improved 1500-day (4.1-year) survival compared with those admitted during the control period (Figure 1). At 1500 days there were 381 deaths in the control period and 303 deaths in the MET period. Thus, the 1500-day rates of survival for patients admitted during the MET and control periods were 71.6% and 65.8%, respectively (log-rank test *P *= 0.001). The odds ratio of death at 1500 days during the MET period was 0.77 (95% confidence interval 0.64–0.92; *P *= 0.004) compared with the control period. This survival benefit was seen for each yearly interval (Table 4).

**Table 4:** Analysis of survival difference between MET and control period for patients undergoing major surgery

	Hospital discharge^a^	1 year	2 years	3 years	4.1 years
Deaths control period	73	195	271	337	382
Deaths MET period	45	133	189	239	303
OR for death	0.57	0.68	0.70	0.71	0.77
95% CI	0.39–0.84	0.55–0.85	0.58–0.84	0.60–0.84	0.64–0.91
*P *value	0.004	0.001	<0.001	<0.001	0.003

^a^As per original publication [10]. CI, confidence interval; MET, medical emergency team; OR, odds ratio.

### Analysis of factors contributing to death

Multivariable analysis of data for patients in both the MET and control periods revealed a number of independent predictors of death (Table 5).

**Table 5:** Multivariate logistic regression analysis for death within 1500 days after surgery for patients admitted in both the control and MET periods

	Odds ratio	95% CI	*P *value	VIF
Age (per 10 years)	1.73	1.61–1.86	<0.001	1.09
MET period^a^	0.74	0.60–0.92	0.005	1.02
Female sex	0.72	0.58–0.89	0.002	1.05
Nonscheduled surgery	1.51	1.21–1.89	<0.001	1.18
Admitting unit				
Cardiac surgery	0.33	0.22–0.48	<0.001	1.28
Thoracic surgery	2.76	1.89–4.03	<0.001	1.36
Orthopaedic surgery	0.50	0.37–0.69	<0.001	1.43
Vascular surgery	0.66	0.46–0.94	0.023	1.20
Neurosurgery	1.60	1.03–2.48	0.036	1.63
Liver transplant unit	0.30	0.09–1.03	0.056	1.05
Oncology	10.48	1.2–91.65	0.034	1.01
General medicine	3.00	1.13–7.95	0.027	1.02
Procedure				
Bronchoscopy	3.13	1.24–7.88	0.015	1.16
Breast surgery	0.20	0.04–0.95	0.043	1.04
Spinal fusion	0.34	0.17–0.68	0.002	1.21
Cerebral resection	3.30	1.6–6.77	0.001	1.33
Cystoscopy ± bladder tumour resection	2.90	1.04–8.08	0.042	1.02

The characteristics of the operation clusters are outlined in Table 1. For this model, the Hosmer-Lemeshow goodness-of-fit statistic was 13.1 (*P *= 0.11). The percentage of appropriately classified patients in the final model is 75.3%. ^a^The OR and 95% CI for introduction of the MET pertains to that derived from the multivariate model. CI, confidence interval; OR, odds ratio; VIF, variance inflation factor.

Increasing age, nonscheduled surgery and admission under units for thoracic surgery, neurosurgery, oncology and general medicine were all independent risk factors for death at 1500 days. Similarly, the operation clusters of bronchoscopy, cerebral resection and cystoscopy (with/without bladder resection of tumour) were independently associated with increased risk for death at 1500 days.

Female sex and admission under units for cardiac surgery, vascular surgery and the liver transplant unit were independently associated with decreased risk for death at 1500 days, as were the operation clusters of breast surgery and spinal fusion. After adjusting for other confounding factors, admission during the MET period was an independent predictor of survival at 1500 days (multivariate odds ratio 0.74, 95% confidence interval 0.60–0.92; *P *= 0.005).

## Discussion

We conducted a follow-up study to assess the long-term survival of the original cohort of patients undergoing major surgery at our hospital [10]. In addition, we assessed the effect of the introduction of a MET service on the outcome of such patients in comparison with a cohort treated before its introduction. Finally, we assessed for possible confounders that might explain the observed differences (mortality, hospital length of stay and SAEs) of our original publication dealing with short-term outcomes [10]. We found that at 1500 days there was a significant 5.8% absolute decrease in long-term mortality among patients treated during the MET period, and that admission during the MET period was an independent predictor of 1500-day survival.

A previous study [6] of 1,125 patients undergoing major surgery (defined as surgery requiring admission for > 48 hours) in our hospital revealed that 16.9% suffered SAEs and 7.1% died. In addition, age above 75 years and unscheduled surgery were predictors of increased risk for in-hospital death. In the present study we confirm that these variables also adversely affect survival to 1500 days.

Subsequently, we demonstrated that the introduction of a MET service was associated with a relative risk reduction (RRR) for postoperative hospital mortality of 36.6%, as well as reductions in ICU admissions (RRR 44.4%) and SAEs (RRR 59.5%) [10]. However, this study did not assess for possible confounders that might have influenced the observed differences in outcome, and its findings were accordingly the subject of criticism. In the present study we have shown that a difference in patient mortality was sustained to a period of 1500 days, and that on day 1500 the odds ratio of death for patients admitted during the MET period was 0.77 compared with the control period (RRR 23%).

Our study revealed that there were a number of differences in the patient cohorts in the MET and control periods. We also identified a number of factors that were independent predictors of increased risk for long-term death, including increasing patient age, male sex, unscheduled surgery and admission under thoracic surgery, neurosurgery, oncology, or general medicine. After adjustment for all assessable confounders, we found that admission during the MET period was associated with a statistically reduced chance of long-term death when compared with admission during the control period. If this effect could be reproduced elsewhere, then the public health consequences would be important. Indeed, in response to preliminary findings that the MET approach may benefit hospital patients, the Institute for Health Improvement has launched a nationwide initiative to introduce such teams in many American hospitals [13]. Thus, knowing whether the putative in-hospital benefits achieved with such teams translate into long-term advantages might be crucial in justifying and sustaining the impetus of such a campaign.

The reduction in mortality between the two study cohorts is perhaps greater than would be expected from the absolute number of MET reviews. We believe that education of hospital staff and the cultural change accompanying the MET is likely to be a substantial contributor to the observed differences in patient outcome associated with the introduction of the MET service.

Our study has several strengths, including a prospective design; verifiable, independent and robust outcome; evidence of a clear effect both before and after adjustment for confounding variables; and a suitable rate of intervention by MET. However, it also has important limitations. First, it was neither double blinded nor placebo controlled or randomized. However, it is not possible to achieve double blinding to intervention by a MET in a single-centre study. Furthermore, introducing a 'sham' intervention as a placebo was considered ethically untenable.

The second limitation is that our analysis revealed differences in characteristics of the patient cohorts admitted during the control and MET periods. However, the beneficial effect of the introduction of the MET service on the long-term outcome of the patients persisted even after adjustment for multiple factors. Nonetheless, we cannot account for other factors that were not assessed but might also have affected patient outcomes. Such factors, rather than the introduction of the MET, might explain our findings.

Our multivariate analysis also identified a number of conditions and surgical procedures that were independent predictors of long-term mortality. It is likely that these differences are due to the prognosis of the underling condition (for example, admission under oncology, general medicine, or neurosurgery). We are unable to comment as to whether the introduction of the MET service was associated with improved outcomes from these conditions. Further work is required to determine whether these conditions or procedures are associated with increased incidence of MET criteria and conditions for which the MET could intervene. We are also unable to comment on the effect of seasonal variation on the differences in observed patient mortality between the two study periods. However, our analysis did identify differences in baseline characteristics of the surgical conditions performed, and yet the benefits of the MET persisted even after adjustment for these differences.

The third limitation of our study is that it demonstrates findings in a single institution only in a particular country. Its findings might not apply to other hospitals or health care systems. However, our institution has all the organizational, structural, logistic and clinical performance features of a typical tertiary referral hospital in a developed country. Nonetheless, it is important to note that the Medical Early Response Intervention and Therapy (MERIT) Study, a cluster multicentre randomized controlled trial of the introduction of the MET in 23 hospitals in Australia, failed, on direct comparison, to show a significant benefit of METs on several important outcomes [14]. A number of differences exist between the MERIT study and our study that may explain these findings. First, the MERIT study did not focus on mortality among patients undergoing major surgery. Second, because of the large hospital-to-hospital variability and the limited number of centres, the study was statistically underpowered. Third, the 'dose' of MET calls in our study (52/1067 = 48.7 METs/1,000 patients) was 5.6 times that of the MERIT study (8.7 emergency calls/1,000 admissions). This difference in MET use may be explained by the longer education and preparation period for our study (12 months) compared with the MERIT study (4 months).

Finally, our study demonstrates reduction in long-term mortality for surgical patients only. The effect on medical patients was not assessed. During the study period there were more than 8,000 medical admissions, making analysis of differences in baseline characteristics and admission diagnosis exceedingly complex. In addition, it is our clinical observation that medical patients have many more chronic comorbidities and fewer acute physiological derangements amenable to intervention and correction by the MET. Nonetheless, we have previously demonstrated that introduction of the MET service was associated with a reduction in the incidence of cardiac arrests in medical patients [11].

## Conclusion

Introduction of an ICU-based MET was associated with an improvement in the long-term outcome of patients undergoing major surgery in a tertiary hospital. Similar studies of long-term outcome for surgical patients from other institutions or health care systems considering the introduction of rapid response systems are now needed to confirm or refute our observations.

## Key messages

• Adverse events are common after surgery and include cardiac arrests and unexpected deaths.

• Implementation of a MET might decrease their incidence and improve survival in surgical patients. However, all studies of METs have been short term.

• We conducted a long-term outcome study to 1500 days postoperatively.

• The odds ratio of death at 1500 days in the MET period was 0.77 when compared with the control period. We found that admission after implementation of a MET was also an independent predictor of decreased 1500-day mortality.

• In our hospital, implementation of a MET was associated with a long-term decrease in mortality in patients undergoing major surgery.

## Abbreviations

ICU = intensive care unit; MET = medical emergency team; SAE = serious adverse event; RRR = relative risk reduction.

## Competing interests

No author has any conflict of interest to declare in relation to this study. The authors certify that all their affiliations with or financial involvement, within the past five years and foreseeable future (for instance, employment, consultancies, honoraria, stock ownership or options, expert testimony, grants or patents received or pending, or royalties) with any organization or entity with a financial interest in or financial conflict with the subject matter or materials discussed in the manuscript are completely disclosed.

## Authors' contributions

The study was conceived, designed and implemented by RB and DG. DG and RB obtained data on long-term outcomes from the Australian Registry of Deaths. Data analysis and compilation of tables was undertaken by DJ. ME conducted the statistical analysis. DJ, ME and RB wrote the manuscript. The final draft of the manuscript was reviewed and approved by all authors.
